# Experimental Investigation of the Effect of Nanofluid Utilization on Heat Transfer Performance in Unmanned Aircraft Radiators with Various Spring-Type Fins

**DOI:** 10.3390/nano15070489

**Published:** 2025-03-25

**Authors:** Beytullah Erdoğan, Abdulsamed Güneş, Gülşah Çakmak

**Affiliations:** 1 Department of Mechanical Engineering, Zonguldak Bülent Ecevit University, Zonguldak 67100, Turkey; beytullah.erdogan@beun.edu.tr; 2 Department of Electric and Energy, Firat University, Elazığ 23119, Turkey; 3 Department of Mechanical Engineering, Firat University, Elazığ 23119, Turkey; gulcakmak@firat.edu.tr

**Keywords:** radiator, unmanned aerial vehicle, nanofluid, fin, spring structure, heat transfer

## Abstract

In the study conducted for the cooling systems of MALE class unmanned aerial vehicles using internal combustion engines, new type radiators were designed using spring-structure fins. Among the radiators formed with spring structures acting as fins, the radiator developed using springs with a pitch of 2.25 mm was named Radiator-Y1, the radiator developed using springs with a pitch of 4.25 mm was named Radiator-Y2, and the radiator developed using springs with a pitch of 8.25 mm was named Radiator-Y3. This design change is seen as an innovative method that can increase heat transfer on the radiator surface and increase cooling performance by increasing the turbulence effect of the air affecting the radiator. Experimental studies were carried out using single type (Al_2_O_3_ and ZnO) and hybrid (ZnO-CuO) nanofluids in addition to pure water. Experiments were carried out using different air speeds (8–10–12 m/s) and different coolant flow rates (20–22 L/min) and radiator performance was investigated. The effects of the surface area created by the spring structure and the turbulence effect on heat transfer were evaluated. As a result of the studies, Radiator-Y1 showed the best cooling performance among the radiators developed with spring structures, followed by Radiator-Y2 and Radiator-Y3. It was observed that the nanofluids used had a positive effect on the cooling performance compared with pure water, as did the hybrid nanofluid compared when compared with single type nanofluids.

## 1. Introduction

In unmanned aerial vehicles powered by internal combustion engines, the main factors affecting the engine cooling performance, especially during landing, takeoff and low altitude flights, are the radiator and coolant fluids used. The coolant in these systems is called internal flow, and the air acting on the radiator is called external flow. These factors increase the cooling performance of the system and ensure that the engine operates under optimum conditions.

Commercial and military use of unmanned aerial vehicles is rapidly increasing worldwide and in our country. The importance of cameras, laser scanning devices and ammunition, called payloads in these systems, has become at least as important as the unmanned aerial vehicle itself. As a result of these effects, the high accuracy, time saving and low-cost flight of these systems is in a critical position. The engine performance in these systems directly affects accuracy and flight duration. Cooling the engine is a very critical issue in unmanned aerial vehicles during landing, takeoff and low altitude operational flights. In the study, experiments were carried out on the radiator and coolant fluids to increase the cooling performance of unmanned aerial vehicles in these important positions.

Fuel consumption can be reduced by increasing cooling performance and therefore energy efficiency, and this results in two positive situations. The first of these is to increase the amount of payload and the second is to extend the flight duration.

In line with this goal, a radiator with a spring-structured fin system was developed as an external flow study in the radiator used in unmanned aerial vehicles. A different study was attempted with regard to cooling systems with these new design radiators. The reason for using springs is the potential to increase the turbulence effect of the air in the external flow affecting the radiator. The second study was conducted with the addition of nano particles to the coolant as an internal flow study. This study was carried out in order to observe how the use of different coolants in systems with new design radiators will affect heat transfer.

In the literature study, no study was found on the use of nanofluids in internal combustion and other types of unmanned aerial vehicle cooling systems. However, there are experimental and theoretical studies related to internal combustion engines and other thermal cycle devices.

Thermodynamic performance was measured in solar energy production systems using eight types of ternary hybrid nano (ZnO-Al_2_O_3_-SiO_2_, Fe-Cu-Ag, Fe-Cu-ZnO, Fe-Cu-MWCNT, Ag-Al_2_O_3_-TiO_2_, MWCNT-Al_2_O_3_-TiO_2_, Fe-MWCNT-TiO_2_ and Fe-Graphene-TiO_2_) fluids by Adun et al. It was recorded that the system showed optimum energy and exergy efficiency and the highest heat storage capacity in the MWCNT-Al_2_O_3_-TiO_2_ mixture [1].

In their study, Sekhar et al. reported that the heat transfer coefficient of Al_2_O_3_ nanofluid, which has a volumetric concentration of 1%, increased by 83% compared with traditional base fluids. In this study, an internal combustion engine with similar characteristics in terms of engine volume and other features was used as an experimental setup with unmanned aerial vehicles [2].

Bozorgan et al. found a 10% increase in heat transfer coefficient and a 24% increase in pumping power compared with the traditional base fluid by using 2% concentration CuO nanofluid in an internal combustion engine radiator [3].

Madhukesh et al. investigated the thermal performance in cylinder plate systems using ternary hybrid nanofluid. It was stated that the heat dissipation rate in the cylinder increased from 2.08% to 2.32% [4]. Similarly, the use of CNT-Fe_3_O_4_/H_2_O hybrid nanofluid provided significant increases in heat transport properties [5]. It was stated that the thermal conductivity of the TiO_2_ and SiO_2_ hybrid nanofluid mixture was increased by up to 22.8% and the dynamic viscosity by up to 62.5% [6].

Koçak et al. investigated the heat transfer performance of two types of nanofluids in automobile radiators. It was determined that 0.3% Ag-doped TiO_2_ nanofluids increased the convection heat transfer coefficient by 26.15% and 27.72% at 1% and 2% concentrations, respectively [7].

Asadi et al. evaluated the heat transfer efficiency in energy management applications using an MWCNT-Al_2_O_3_ hybrid nanofluid at 0.125–1.5 volumetric concentration ratios and temperature range of 25–50 °C. It was revealed that dynamic viscosity and thermal conductivity increased with the increase in concentration ratio [8].

Al₂O₃-CuO hybrid nanofluid increases the heat transfer rate by more than 10% compared with basic fluids [9]. Jilte et al. reported that the use of hybrid nanoparticles in cylindrical Li-Ion thermal cells, for which they performed CFD analysis, increased the cooling performance [10].

Bhogare et al., in an experiment using 50% ethylene glycol-water based nanofluid containing 1% Al_2_O_3_ nanoparticles in a radiator similar to the one used in UAVs, found a 40% increase in heat transfer coefficient [11]. Ambreen and Saleem et al., in their experimental and numerical analyses using MXene nanofluid to increase thermal efficiency in basic heat transfer processes, found that the average Nusselt number and pumping power of the nanofluid used increased. It was stated that the volumetric concentration ratio of the nanofluid used was related to the pressure drop and thermal performance [12].

Nirnjalkumar et al., in their experimental study with Al_2_O_3_ nanofluids at 0.25% and 0.5% volumetric concentration, observed an average of 50% improvement in the heat transfer rate of the system [13].

Duangthongsuk and Wongwises heat transfer performance, pressure drop, cooling performance and pumping power analysis were performed with the use of SiO_2_ nanofluid in heat sinks with different fin structures such as circular (MCFHS) and square (MSFHS). It was stated that the values increased as desired in the use of nanofluid, and the square section fin structure showed more pressure drop than the circular one [14].

Topuz et al. investigated the thermal performance of Al_2_O_3_/water, ZnO/water and TiO_2_/water nanofluids on microchannel structures at 0.5%, 0.7% and 1% volumetric concentration rates. It was stated that Al_2_O_3_/water nanofluid exhibited the best thermal performance compared with the others and this improvement was dependent on the stability of the nanofluid; the stability of Al_2_O_3_/water was observed as 2 months, ZnO/water as 7 days and TiO_2_/water as 21 days [15].

In an experimental study in which the same ratio of Al_2_O_3_/water nanofluid and ethylene glycol mixture was used in an automobile radiator, Topuz et al. evaluated the heat transfer rate and pressure losses of the radiator and stated that significant improvements were achieved in cooling performance [16].

In the study by Wang et al., in which they used ternary hybrid nanofluid (Al_2_O_3_-Cu-TiO_2_) to measure the amount of heat transfer occurring in heat pipes in thermosyphons, it was determined that the heat transfer rate increased by 22.5% [17]. Al_2_O_3_-SiO_3_/water nanofluid required 95% more pumping power than Al_2_O_3_-SiO_2_/water nanofluid [18].

It has been stated that in cooling an elastic plate using ternary nanofluid, the Nusselt number increases up to 42% and the cooling performance increases up to 4.5% under various conditions [19]. It is stated that ternary nanofluid formed with aluminum, copper and nickel nanoparticles has higher Nusselt number calculations compared with linear and nonlinear radiative flows in opposing flows [20].

Kumar et al. stated that the optimum mixing ratio for properties such as heat transfer coefficient and pressure drop in the use of Al_2_O_3-_MWCNT hybrid nanofluid is 3:2 [21].

In the study conducted by Kumar, Chand and Hassan, the use of MWCNT-SiO_2_ hybrid nanofluid in a vehicle radiator with louver type fins provided a cooling power of around 40% compared with the use of ethylene glycol-water [22].

Kumar, Hassan and Chand observed that the heat transfer coefficient increased by 42.5%, 47.4% and 51.1% with the use of Al_2_O_3_, CuO and ZnO nanofluids in louvered finned vehicle radiators [23].

While investigating the effect of fin shape and nanoparticle usage in compact finned tube heat exchanger automobile radiators, it was stated that louver type finned radiator provided 24.6% heat transfer increase compared with flat finned radiator and this rate increased even more with the addition of Al_2_O_3_ and CuO nanoparticles [24].

It was stated that the pressure drop in the exchanger with louver type fin structure was reduced up to 24.2% especially in the horizontal louver finned heat exchanger by redesigning the louver edges in different shapes and sizes as horizontal, vertical and inclined [25].

Subhedar et al. reported in their study that the use of Al_2_O_3_ nanofluid containing 50% water and mono ethylene glycol mixture at 0.2% volumetric concentration provided a 30% increase in the heat transfer of the radiator and that the radiator could reduce fuel consumption with aerodynamic improvement [26].

In their experimental study with Cu nanoparticles, Lavate et al. found an increase of approximately 45% in the heat transfer performance of the system compared with the base fluid with the use of nanofluid [27]. Similarly, Ravisankar reported that they observed an average heat transfer coefficient increase ranging from 16% to 31% with CuO nanofluid in internal combustion engine radiators with nanoparticle concentration rates between 0.0025% and 0.05%. It was also determined that this increase was directly proportional to the volumetric flow rate of the coolant and the air speed [28]. Madhesh and Kalaiselvam stated that the use of Cu- and TiO_2_-doped nanofluids in tube heat exchangers provides 48.4% higher heat transfer coefficient compared with water due to the increase in thermal conductivity and kinetic diffusion mobility [29].

Moghadassi et al., in their study using an Al_2_O_3_-Cu hybrid nanofluid, stated that the nanofluid provided a higher Nusselt number and heat transfer coefficient compared with traditional coolants and that the increase in volumetric density in nanofluids also increased the pressure drop [30]. Erdoğan et al., in their study on radiators with louver type fin structures, in which they examined the effects of the use of nanofluid in cooling systems of internal combustion engines, stated that the use of nanofluid increased the heat transfer performance by 9.5%, with a 4 m/s air speed and a 10 L/min coolant flow rate and led to an increase in pump power [31].

Palaniappan and Ramasamy, in their study on hybrid nanofluids that were prepared using various nanoparticles, stated that they achieved a 21% increase in heat transfer coefficient compared with the base fluid in internal combustion engine cooling systems at 2% volumetric concentration and an 8000 Re number [32].

Elsaid tested the nanofluids prepared with Co_3_O_4_ and Al_2_O_3_ at concentrations of 0.02, 0.05, 0.1 and 0.2% in a vehicle radiator and concluded that the cobalt Co_3_O_4_ nanofluid showed higher thermal performance than the Al_2_O_3_ nanofluid and that the addition of ethylene glycol increased the pumping power by reducing the Nusselt number [33].

Ben-Mansoor and Habib, Rostamani et al., and Bianco et al. developed new formulations to determine the density, specific heat and heat transfer coefficient of hybrid nanofluids through their experimental studies [34,35,36].

Sajid and Ali stated in their study that the thermal conductivity of hybrid nanofluid combinations is directly dependent on the stability factor. Surfactants and pH control are the factors affecting this stability [37]. It is suggested that the thermal conductivity in nanofluids used as hybrids is higher than the thermal conductivity of individual nanofluids in the mixture [38,39,40,41,42,43,44,45,46]. However, it has been found that the thermal conductivity of some nanofluids in the hybrid state is lower than their individual state thermal conductivity due to the incompatibility of the particles [47,48].

Pathak et al. have stated that nanoparticle coating methods also enhance magnetic properties [49]. In their study, they incorporated a magnetic fluid layer into annular finned tube heat exchangers to improve heat transfer. This approach increased system efficiency by more than 20%, while optimizing the annular fin geometry resulted in a 35% enhancement in heat transfer [50].

Elsebay et al. have stated that adding Al_2_O_3_ and CuO nanoparticles to the coolant increased the cooling performance, but that negative effects were observed when a certain ratio was exceeded. Cooling performance was increased by 45% and 38% with Al_2_O_3_ and CuO nanoparticles, respectively [51]. Similarly, Arif et al., who worked with Al_2_O_3_ and CuO nanoparticles, stated that the hybrid fluid showed higher cooling performance compared with the single-type and hybrid nanofluids they made using these particles [52].

Sahoo achieved 19.35% and 7.2% heat transfer performance increase at 3% and 1% concentrations, respectively, by using hybrid nanofluids containing spherical CuO nanoparticles in internal combustion engine radiators [53]. However, in another study, Sahoo also stated that these nanofluids caused pressure drop and required higher pumping power [54].

Gareyev et al., who conducted studies on increasing flight duration and efficiency on unmanned aerial vehicles, determined 14 different criteria affecting flight performance and concluded through their analyses that the weight criterion in aircraft is the most effective factor on aircraft performance. They stated that the weight factor in aircraft is important not only during landing and takeoff but also throughout the entire flight duration [55].

There is no study in the literature on the cooling system of a real-scale UAV. In the existing literature, there are studies on internal flow or only external flow regarding radiators of internal combustion engine vehicles. In this study, both external and internal flow conditions were investigated in a real UAV radiator with a geometry of 330 mm × 125 mm × 34 mm, which corresponds to a radiator used in MALE class unmanned aerial vehicles. In the external flow analysis, the cooling performance was evaluated and compared with the existing radiator by using three different and new fin types in the radiator. In the internal flow analysis, the cooling performance was investigated and compared with the existing coolant by using single and hybrid nanofluids as coolant. The experiments were carried out under the conditions that the coolant temperature was 70 °C, the fluid flow rate was 20–22 L/min, and the air speeds were 8, 10, and 12 m/s, respectively. The results obtained in this study provide important information to ensure flight safety by increasing the cooling performance of unmanned aerial vehicle radiators in the most efficient way.

## 2. Materials and Methods

In this section, the design of the experimental setup used to simulate the unmanned aerial vehicle cooling system, the types of radiators used, the process of using nanofluids to be used in these radiators and the applied methodology are explained.

### 2.1. Nanofluid Preparation

In the experimental study, nanofluids were prepared by a two-stage method. Pure water was used as the base fluid, and in addition to 0.3 vol.% Al_2_O_3_, 0.3 vol.% ZnO and 0.15 vol.% hybrid ZnO-CuO nanofluids were preferred. The selected nanoparticles were selected from those with high thermal conductivity and stability properties in the literature. Nanofluids were prepared by mixing the nanoparticles measured with a precision balance with pure water for 15 min with a magnetic stirrer and 30 min with an ultrasonic homogenizer (CY-500, power: 500 W, frequency: 20 kHz, probe diameter/length: Ø5, 6/60 mm). At least 5 L of nanofluid was produced for each experiment. Thermal properties are presented in Table 1.

### 2.2. Thermal Conductivity Measurements

**Figure 1:**
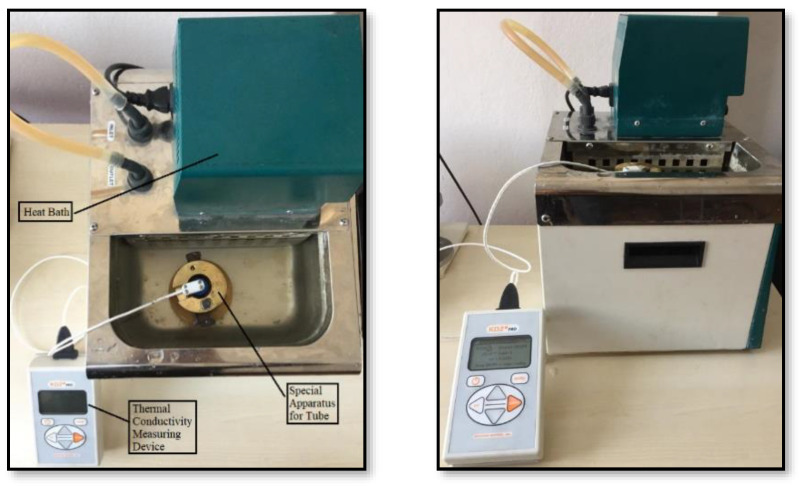
General application steps of the method.

The thermal conductivity measurements of the nanofluids given in Table 1 were carried out using a Decagon KD2 Pro (METER Group, Inc. USA, Pullman, WA, USA) thermal conductivity measuring device with the measurement range of 0.02–2.00 W/m.K and ± 5% accuracy, specified in Figure 1, together with a KS-1 sensor (Robert Bosch GmbH Robert-Bosch-Platz 1, Gerlingen-Schillerhöhe, Germany). Thermal conductivity values at different temperatures were measured by immersing the tubes containing the nanofluid in a heat bath set to the desired temperature, using a special apparatus that ensures that they remain fixed. The measurements were repeated at least three times for each temperature and the average values were obtained.

### 2.3. Viscosity Measurements

**Figure 2:**
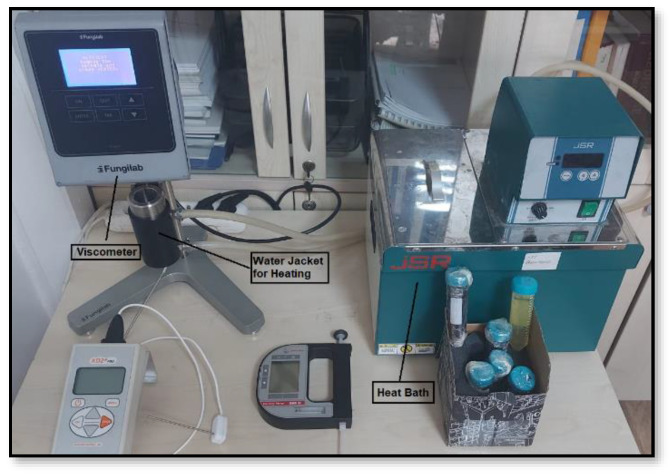
Dynamic viscosity measurement process.

Dynamic viscosity measurements of nanofluids were made with a Fungilab Smart L (Fungilab S.A., Sant Feliu de Llobregat, Spain) device (±2%) with a measurement range of 0–2000 mPa.s as given in Figure 2. A low viscosity adapter was used in the viscosity measurements of nanofluids prepared as a minimum 16 mL. Dynamic viscosity values were measured at the desired temperatures by repeating them at least 3 times with the help of a heat bath connected to the water jacket in the viscosity measuring device.

### 2.4. Density Measurements

**Figure 3:**
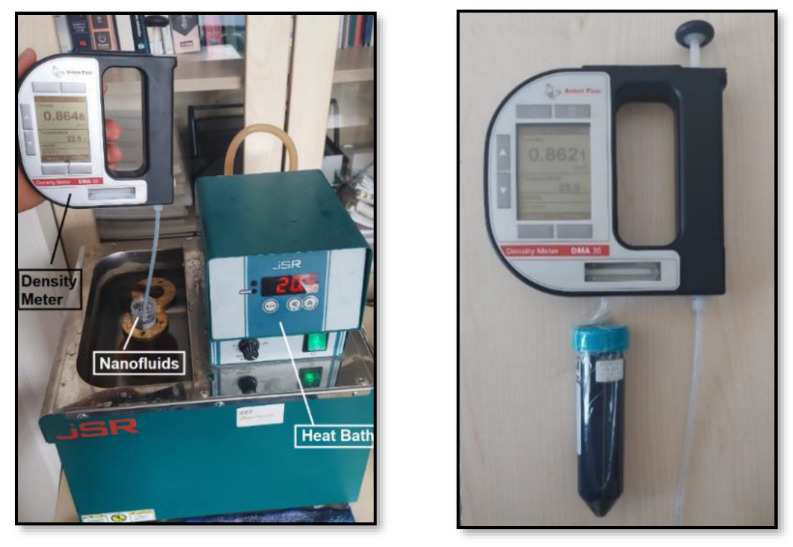
Density measurement process.

The densities of nanofluids prepared at different concentration ratios were measured with an Anton Paar Density Meter DMA35 (Anton Paar GmbH, Graz, Austria) device with the measurement range of 0–3 g/cm^3^ and ±1% precision, specified in Figure 3. Density measurements at different temperatures were carried out with 2 mL samples taken from tubes immersed in a heat bath. The measurements were repeated at least three times for each temperature and the average values were obtained.

### 2.5. Specific Heat Measurements

As a specific heat measurement device was not available, the specific heat capacities of the nanofluids used in the experiments were calculated using the commonly employed thermal equilibrium model in the literature, as given by Equation (1) [56].

(1) $$c_{nf}=\left[\rho_{np}c_{np}\emptyset +\rho_{bf}c_{bf}(1-\emptyset )\right]/\rho_{nf}$$

Thermal conductivity, dynamic viscosity and density values of the prepared nanofluids were measured at ambient temperature (T = 20–22 °C) and are given in Table 1.

### 2.6. Conducting the Experiments

**Figure 4:**
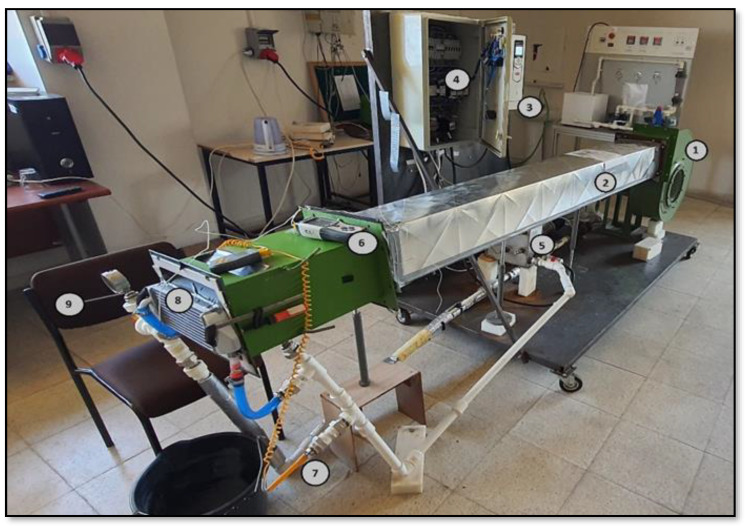
Experimental setup; (1) fan, (2) duct, (3) frequency converter, (4) control panel, (5) fluid tank, (6) air velocity measurement point, (7) coolant temperature measurement point, (8) radiator, and (9) coolant pressure measurement point.

**Figure 5:**
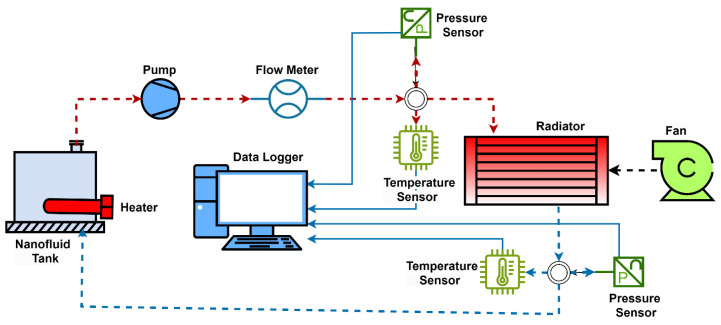
Experimental setup schematic representation.

**Figure 6:**
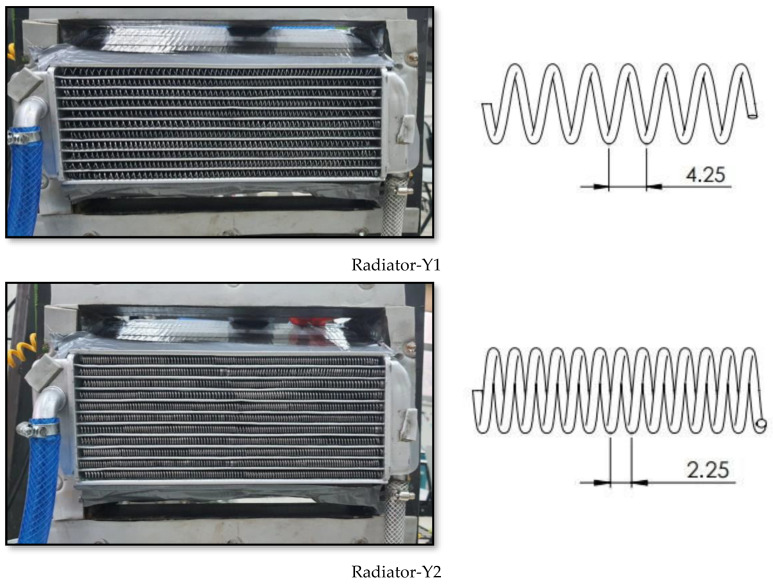

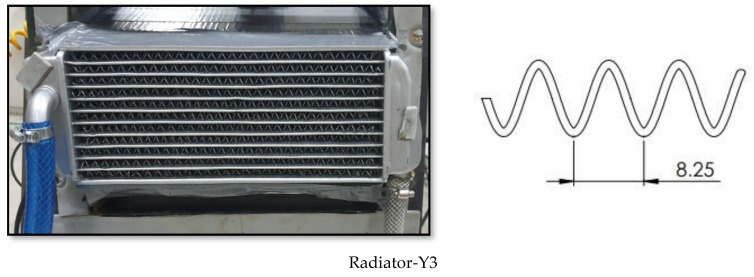
Improved Radiator-Y1, Radiator-Y2 and Radiator-Y3 visuals and spring fin structures.

The images of the experimental setup established to determine and compare the cooling performances of the radiators designed to be used in UAVs are presented in Figure 4. The schematic representation of the experimental setup is given in Figure 5. The images of Radiator-Y1, Radiator-Y2 and Radiator-Y3, whose fin structures were redesigned, are given in Figure 6. The technical specifications of the radiators used are given in Table 2. In the experiments, the inlet and outlet temperatures of the coolant fluids were continuously measured and recorded by digital thermometers (Brand: TASI 8620, measuring range: −50–1350 °C, accuracy: ±0.3% rdg + 1 °C in 50–1000 °C), and the inlet and outlet pressures were measured and recorded by manometers (Brand: Pakkens, measuring range: 0–40 bar, accuracy: ±%0.5 FS in 25 °C). Experiments were carried out by measuring the air velocity acting on the radiators using an anemometer (Brand: Airflow TA 2, measuring range: 0–30 m/s; accuracy: velocity: ±3% of reading ± 1 digit or ± 0.06 m/s ± 1 digit whichever is the greater; flow rate: ±3% of reading ± 1 digit or ±(0.06 × input area) m^3^/s ± 1 digit whichever is the greater) and the coolant flow rate was measured using a flow meter (Brand: ABB, Q_max_: 45 L/min, T_max_: 130 °C, accuracy: %0.4). The experiments were carried out under the same internal and external flow conditions by mounting the radiators given in Figure 6 to the experimental setup. In addition, 100% pure water, 0.3% volumetric concentration Al_2_O_3_, 0.3% volumetric concentration ZnO and 0.15% volumetric concentration hybrid ZnO-CuO nanofluids were used as internal flows in the radiators in question. In each experiment, air velocity, coolant flow rate, coolant inlet and outlet temperatures and pressure values were recorded.

At high altitudes, the need for cooling systems decreases due to extremely low ambient temperatures. The experimental setup was designed by considering conditions in which cooling systems operate actively, taking into account the varying altitudes and external temperature fluctuations encountered by real unmanned aerial vehicle systems. The coolants in the cooling system of a real unmanned aerial vehicle (Turkish Aerospace Industries, Ankara, Türkiye) used in the system are heated to the desired temperature by means of heating resistors in the fluid tank and are transmitted to the radiator by a high-temperature pump. The flow rate of the coolant sent to the system is precisely controlled by a throttle valve, and the fluid flow rate is adjusted in accordance with the real operating conditions. The air speed acting on the radiator is adjusted to the air speed conditions during flight, takeoff and landing on the UAV radiator using a fan whose fan speed can be adjusted by a frequency converter, and all experiments are carried out under these conditions.

In the new design radiators, the radiator in which the springs with a pitch of 4.25 mm are used as fins is called Radiator-Y1, the radiator in which the springs with a pitch of 2.25 mm are used as fins is called Radiator-Y2, and the radiator in which the springs with a pitch of 8.25 mm are used as fins is called Radiator-Y3. In each channel, the specified springs are soldered and assembled in three consecutive positions. There are a total of 33 springs along the radiator channel in each radiator. The real images of these radiators and the details of the fin and spring structures used are presented below.

### 2.7. Heat Tansfer Analyis

In the hydrodynamic analyses performed in the studies, whether the flow is laminar or turbulent is determined by calculating the Reynolds number (Re) with the equation in (2) [57]. This calculation was carried out for the flows in pipes and channels using the coolant flow rate in the system and the air velocity acting on the radiators.

(2) Re=u Dh v=Internal ForcesViscous Forces

The dimensionless Prandtl number (Pr), which expresses the coupled change of the hydrodynamic and thermal boundary layers, is defined as the ratio of momentum dissipation to thermal dissipation [57]. The Pr number is calculated by Equation (3).

(3) $$\mathrm{Pr}=\frac{\upsilon }{\alpha }=\frac{Momentum\;Dissipation}{Thermal\;Dissipation}$$

The thermal diffusivity coefficient α is expressed as k/*ρ*cp. Convection heat transfer in pipes and channels is calculated with the Nusselt number (Nu) [57]. The Nu number is used to determine the heat convection coefficient and depends on the channel geometry with the Re and Pr numbers. In other words, it expresses the ratio of heat convection to heat transfer by conduction and is calculated with Equation (4). The hydraulic diameter (D_h_) calculation is calculated with Equation (5).

(4) $$\mathrm{Nu}=\frac{hD_{h}}{k}=\frac{Heat\;transfer\;by\;convection}{Heat\;transfer\;by\;conduction}$$

(5) $$D_{h}=\frac{4\;x\;Area}{Wet\;Environment}$$

Equations (6) and (7) were used to calculate the amount of heat transfer occurring in the process [57].

(6) $${\dot{Q}}_{cool}={\dot{m}}_{cool}\times C_{p,cool}\times (T_{cool,in}-T_{cool,out})$$

(7) $${\dot{m}}_{cool}=\rho_{cool}\times V_{cool}\times A$$

Equations (8) and (9) are used in the calculations for the air side [57].

(8) $${\dot{m}}_{air}=\rho_{air}\times V_{air}\times A$$

(9) $${\dot{Q}}_{air}={\dot{m}}_{air}\times C_{p,air}\times (T_{air,in}-T_{air,out})$$

The high thermal conductivity of nanofluids is explained by “Brownian Motion” [58]. Brownian motion refers to the random motion of microscopic particles in the liquid. In heat exchangers, the total heat transfer coefficient is calculated as in Equation (10) by taking into account the convection and conduction resistances [59].

(10) $$\frac{1}{UA}=\frac{1}{U_{hot}A_{hot}}=\frac{1}{U_{cold\;}A_{cold}}=\frac{1}{{\left(hA\right)}_{hot}}+R_{w}+\frac{1}{{\left(hA\right)}_{cold}}$$

When heat transfer analysis is performed on finned, i.e., increased, surfaces, if fins are added to both sides of the surfaces, the formula is expanded a little more and written and calculated as in Equation (11).

(11) $$\frac{1}{UA}=\frac{1}{{\eta_{o}(hA)}_{hot}}+R_{w}+\frac{1}{{\eta_{o}(hA)}_{cold}}$$

The expression η_o_ in the equation represents the surface efficiency (surface effectiveness) and is calculated by determining the efficiency of all fins. The heat transfer equation for the η_o_ value is calculated with the expression given in (12) [59].

(12) $$\dot{Q}=\eta_{o}hA(T_{b}-T_{\infty })$$

The total heat transfer coefficient is calculated by the heat transfer coefficients of the fluids, the fouling factors and the geometrical features. T_b_ indicates the base surface temperature and the surface area and R_w_ represents the wall heat transfer resistance for clean surfaces. Fouling in heat exchangers reduces performance by increasing the heat transfer resistance and creates additional thermal resistance as the fouling factor (R_f_). These factors depend on the operating temperature, fluid flow rate and the life of the heat exchanger [59]. The total heat transfer coefficient is expressed by Equation (13). In this equation, the index h represents the hot fluid and the index c represents the cold fluid.

(13) $$\frac{1}{UA}=\frac{1}{U_{h}A_{h}}=\frac{1}{U_{c\;}A_{c}}=\frac{1}{{\eta_{o}(hA)}_{h}}+\frac{R_{f,h}}{{\eta_{o}(hA)}_{h}}+R_{w}+\frac{1}{{\eta_{o}(hA)}_{c}}+\frac{R_{f,c}}{{\eta_{o}(hA)}_{c}}$$

## 3. Research Findings and Discussion

In this study, three different radiator types redesigned using spring structures for use in unmanned aerial vehicles were used. In the experiments, the cooling performances of the new design radiators were examined. In the new design radiators, four different radiators with fin structures, the details of which are given in Figure 6, and four different coolants, namely pure water, Al₂O₃ nanofluid, ZnO nanofluid and ZnO + CuO hybrid nanofluid, were used, respectively. The experiments were carried out at 20 and 22 L/min fluid flow rates and 8–10–12 m/s air speeds, and the cooling loads were analyzed. When the data obtained as a result of the calculations were examined, it was seen that the use of nanofluid increased the heat transfer performance and revealed the effect of the spring structure on the heat exchanger design. In the experiments, the outlet temperatures of the coolants entering at 70 °C for each radiator and the obtained cooling powers are given in Table 3, Table 4 and Table 5. Detailed evaluations made for each radiator are presented below.

**Figure 7:**
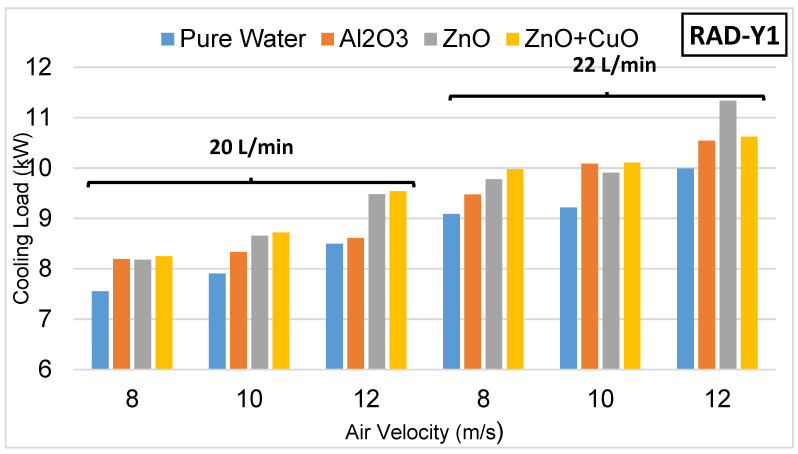
Experimental results with Radiator-Y1.

The experimental results of Radiator-Y1 with medium pitch (4.25 mm) are presented in Figure 7. As Radiator-Y1 offers wider flow channels compared with Radiator-Y2, its flow resistance is lower, which allows it to exhibit a balanced performance in terms of convective heat transfer. Therefore, Radiator-Y1 showed the best cooling performance among the redesigned radiators.

Among the coolants used in Radiator-Y1, ZnO nanofluid achieved the highest heat transfer rate. In most of the other parameters, the performance ranking was determined as ZnO nanofluid and then Al₂O₃ nanofluid, after the use of hybrid nanofluid. Pure water exhibited the lowest heat transfer performance.

Increasing the coolant flow rate from 20 L/min to 22 L/min in Radiator-Y1 increased the cooling load. Radiator-Y1 has a structure that is sensitive to fluid flow rate and also responds to air velocity changes. In particular, a significant heat transfer increase was observed when the air velocity increased from 10 m/s to 12 m/s. At an air velocity of 12 m/s, the highest cooling load was obtained in the experiment in which ZnO nanofluid was used. These findings show that Radiator-Y1 offers a more balanced performance in terms of both air velocity and fluid flow rate and works efficiently, especially at high air velocities. These data show that Radiator-Y1 with a close-pitch-spring structure exhibits limited performance at low air velocities but achieves better results at high air velocities and with nanofluids with high thermal conductivity.

**Figure 8:**
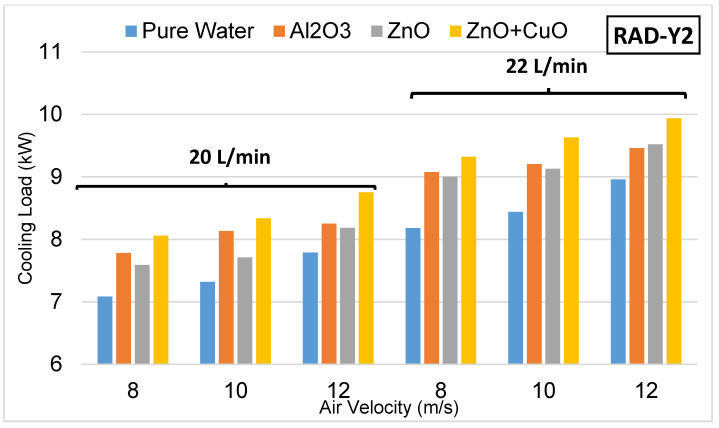
Experimental results with Radiator-Y2.

The results obtained in Radiator-Y2, which was designed using a total of 33 springs with a pitch of 2.25 mm, are presented in Figure 8. In this study, the tight spacing of the springs increases the heat transfer area between the fluid and the surface, providing a more homogeneous heat distribution. However, the external flow passing through narrow channels can increase friction losses, which can increase the flow resistance and have a negative effect on heat transfer.

Among the coolants used in Radiator-Y2, the ZnO + CuO hybrid nanofluid exhibited the highest cooling performance at all air speeds and fluid flow rates. Among the coolants containing a single type of nano particle, ZnO nanofluid showed a better performance compared with Al₂O₃ nanofluid. Pure water had the lowest heat transfer capacity compared with nanofluids. This is due to the fact that nano particles increase the thermal conductivity and increase the convection coefficient.

The heat transfer at a coolant flow rate of 22 L/min has increased significantly due to the higher speed of the fluid circulating on the radiator surface and the resulting increase in the convective heat transfer coefficient.

A significant increase in cooling capacity was observed by increasing the air speed acting on the radiator from 8 m/s to 12 m/s. In particular, the maximum cooling load was achieved by using the ZnO + CuO hybrid nanofluid at 12 m/s air speed.

**Figure 9:**
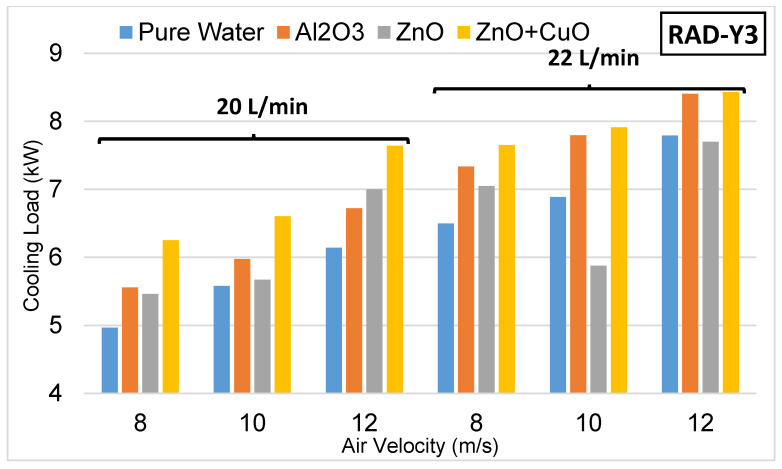
Experimental results with Radiator-Y3.

The results obtained for Radiator-Y3 are presented in Figure 9. As it has the widest pitch range (8.25 mm), it has reduced the turbulence effect, causing the heat transfer area to narrow and providing less resistance to the air flow. The effect of nanofluids used in Radiator-Y3 is parallel to Radiator-Y1 and Radiator-Y2. In addition, the increase in air speed and coolant flow rate has positively affected the heat transfer rate.

**Figure 10:**
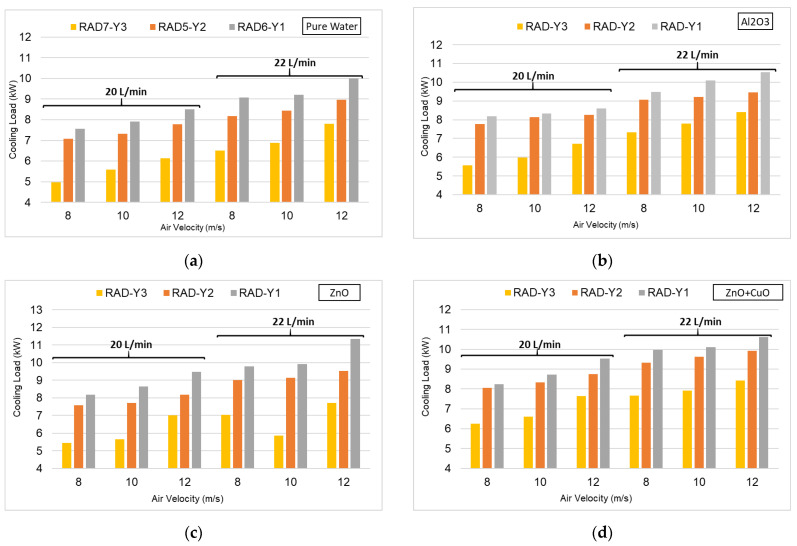
(**a**–**d**) Effect of nanofluids used in experiments on radiators.

In Figure 10, graphs showing the cooling loads occurring in the radiators used for each coolant are presented together. It was observed that, as the air speed increases, the heat transfer increases and, as a result, the cooling load occurring in the process increases. In the comparisons made between the radiators, the highest cooling load belongs to Radiator-Y1. Particularly at 12 m/s air speed and 22 L/min flow rate, Radiator-Y1 exhibited higher performance than the other radiators. Radiator-Y3 showed the lowest performance compared with the other radiators. This situation shows that the wide-pitch-spring structure does not contribute enough to the heat transfer and may create thermal resistance.

The use of nanofluid in the cooling system provided generally better heat transfer compared with pure water. Radiator-Y1, in particular, also showed the best performance at this point. Radiator-Y2 gave good results under certain conditions, while Radiator-Y3 had the lowest performance. In the graphs given in Figure 10b,c, it was observed that ZnO nanofluid provided slightly higher heat transfer compared with Al₂O₃ nanofluid. Under these conditions, Radiator-Y1 showed the best performance. Particularly at 12 m/s air speed and 22 L/min flow rate, the cooling load reached up to 12 kW. Radiator-Y2 showed good performance under certain conditions, while Radiator-Y3 showed the lowest performance. According to Figure 10d, the use of hybrid nanofluid provided the highest heat transfer among all nanofluids. Especially at 12 m/s air speed and 22 L/min flow rate, Radiator-Y1 achieved the highest cooling load. Radiator-Y2 was in second place here, while Radiator-Y3 had the lowest cooling load. The use of hybrid nanofluid shows better performance, especially at high air speeds. When an overall evaluation is made, the radiator that shows the best performance is Radiator-Y1. Radiator-Y2 showed moderate performance in some cases. Radiator-Y3 has the lowest cooling capacity, which may be due to the wide pitch-spring structure not creating sufficient turbulence.

Compared with pure water, nanofluids generally showed better performance. Hybrid ZnO + CuO nanofluid provided the highest cooling load. ZnO nanofluid showed higher performance compared with the Al₂O₃ nanofluid. Hybrid nanofluids are systems containing different types of nanomaterials, and this diversity provides more efficient heat transfer by complementing the heat conduction properties. While one nanomaterial can reduce viscosity, the other can increase the heat conduction coefficient. In addition, the hybrid use of nanomaterials with different thermophysical properties can provide a more homogeneous temperature distribution. Therefore, it is thought that ZnO + CuO hybrid nanofluids offer higher cooling performance compared with mono nanofluids.

## 4. Conclusions

Radiator-Y1, which exhibits the best cooling performance, has kept the heat transfer performance at the highest level by keeping the air flow resistance at reasonable levels and at the same time keeping the surface area in contact with the fluid large enough. By increasing the air speed and fluid flow rate, the highest cooling capacity is achieved. In particular, when ZnO + CuO hybrid nanofluid is used, the maximum cooling load is approximately 11.5 kW at 12 m/s air speed and 22 L/min coolant flow rate.

The balanced flow conditions offered by Radiator-Y1 provided consistent performance at both high and low air speeds. As it has lower flow resistance compared with Radiator-Y2, it increased convective heat transfer by allowing the fluid to contact the radiator surface more effectively. This study reveals that Radiator-Y1 achieves maximum heat transfer by providing an optimum balance on both the air and fluid sides.

It was observed that Radiator-Y1 provided approximately 11.3 kW cooling load at the highest performance conditions, Radiator-Y2 provided approximately 9.8 kW, and Radiator-Y3 provided approximately 8.4 kW cooling load at the highest performance conditions. The obtained data show that the highest cooling capacity was obtained in Radiator-Y1 with medium frequency (4.25 mm pitch).

## 5. Uncertainty Analysis

Uncertainty analysis was performed to increase the reliability of the data obtained in the experiments and to interpret the results with high accuracy. Although the measurement devices are sensitive, there are margins of error, which can make it difficult to calculate the real values. In this study, the error rates of the measurement devices were determined and included in the calculations. Uncertainties on heat transfer and pressure drop were analyzed to evaluate the cooling performance.

Uncertainty analysis is presented in two parts as directly measurable variables and calculable variables. In the analysis performed according to the Kline and McClintock [60] method, the maximum uncertainty in heat transfer was calculated as 26.25% and in pressure drop as 12.73%. These values are presented in Table 6 and Table 7.

## 6. Suggestions

In order to further increase energy efficiency, studies can be conducted on the fin geometries of medium-pitch radiators. Heat transfer performance can be increased by using air flow regulators to increase flow resistance to the desired level. It is anticipated that cooling performance can be further improved with different nanofluid mixtures.

## Figures and Tables

**Table 1:** Thermal properties of nanofluids.

Coolant	Volumetric Concentration Ratio (%)	Density (kg/m^3^)	Specific Heat (J/kgK)	Thermal Conductivity (W/mK)	Dynamic Viscosity (mPa.s)
Pure water	100	998.20	4190.00	0.650	1.002
Al_2_O_3_ Nanofluid	0.3	1003.80	3541.05	0.727	1.170
ZnO Nanofluid	0.3	999.00	3541.44	0.769	1.090
Hybrid (ZnO-CuO) Nanofluid	0.15 ZnO 0.15 CuO	998.20	3528.56	0.651	1.020

**Table 2:** Technical specifications of the radiators.

Features	Value
Brand	Brazing
Width × height × depth	330 mm × 125 mm × 34 mm
Channel dimensions (external)	2.2 mm × 32 mm
Channel wall thickness	0.3
Channel dimensions (internal)	1.6 mm × 32 mm
Number of channels Fin type	10 Spring
Fin pitch	9 fpi
Channel and fin material	Aluminum

**Table 3:** Radiator-Y1 experimental data.

	Fluid Flow Rate	(L/min)	22			20		
	Air Velocity	(m/s)	8	10	12	8	10	12
Radiator-Y1	Pure water	Cooling power (kW)	9.475	10.086	10.545	8.197	8.336	8.614
		Fluid outlet temp. (°C)	63.8	63.4	63.1	64.1	64	63.8
	Al_2_O_3_ Nanofluid	Cooling power (kW)	9.779	9.909	11.337	8.182	8.654	9.480
		Fluid outlet temp. (°C)	62.5	62.4	61.3	63.1	62.7	62
	ZnO Nanofluid	Cooling power (kW)	9.086	9.215	9.991	7.554	7.907	8.495
		Fluid outlet temp. (°C)	63	62.9	62.3	63.6	63.3	62.8
	Hibrit (ZnO-CuO) Nanofluid	Cooling power (kW)	9.978	10.107	10.623	8.249	8.719	9.540
		Fluid outlet temp. (°C)	62.3	62.2	61.8	63	62.6	61.9

**Table 4:** Radiator-Y2 experimental data.

	Fluid Flow Rate	(L/min)	22			20		
	Air Velocity	(m/s)	8	10	12	8	10	12
Radiator-Y2	Pure water	Cooling power (kW)	9.322	9.628	9.934	8.058	8.336	8.753
		Fluid outlet temp. (°C)	63.9	63.7	63.5	64.2	64	63.7
	Al_2_O_3_ Nanofluid	Cooling power	9.000	9.130	9.519	7.591	7.709	8.182
		Fluid outlet temp. (°C)	63.1	63	62.7	63.6	63.5	63.1
	ZnO Nanofluid	Cooling power (kW)	8.181	8.439	8.957	7.084	7.319	7.790
		Fluid outlet temp. (°C)	63.7	63.5	63.1	64	63.8	63.4
	Hybrid (ZnO-CuO) Nanofluid	Cooling power (kW)	9.074	9.203	9.462	7.780	8.132	8.249
		Fluid outlet temp. (°C)	63	62.9	62.7	63.4	63.1	63

**Table 5:** Radiator-Y3 experimental data.

	Fluid Flow Rate	(L/min)	22			20		
	Air Velocity	(m/s)	8	10	12	8	10	12
Radiator-Y3	Pure water	Cooling power (kW)	7.335	7.794	8.405	5.557	5.974	7.641
		Fluid outlet temp. (°C)	65.2	64.9	64.5	66	65.7	64.5
	Al_2_O_3_ Nanofluid	Cooling power (kW)	7.049	5.877	7.700	5.461	5.579	7.000
		Fluid outlet temp. (°C)	64.6	65.5	64.1	65.4	65.3	64.1
	ZnO Nanofluid	Cooling power (kW)	6.497	6.886	7.792	4.963	5.671	6.142
		Fluid outlet temp. (°C)	65	64.7	64	65.8	65.2	64.8
	Hybrid (ZnO-CuO) Nanofluid	Cooling power (kW)	7.653	7.911	8.428	6.252	6.604	6.722
		Fluid outlet temp. (°C)	64.1	63.9	63.5	64.7	64.4	64.3

**Table 6:** Uncertainties of variables measured.

No	Instrument	Range	Variable Measured	Total Uncertainty	Uncertainty	
					Min	Max
1	Temperature sensor	−40 + 125 °C	Fluid inlet temperature, T_in_	$U_{\mathrm{Fixed},T_{\mathrm{in}}}=1.02\;{}^{\circ}C\;U_{T_{\mathrm{in}}}=\sqrt{U_{Fixed,T_{in}}^{2}+U_{Random,T_{in}}^{2}}$ $=\sqrt{1.02^{2}+0^{2}}≅$ ±1.02 °C	1.0726%	1.0759%
2	Temperature sensor	−40 + 125 °C	Fluid outlet temperature, T_out_	$U_{T_{out}}=\sqrt{1.02^{2}+0^{2}}≅$ ±1.02 °C	1.1384%	1.3747%
3	Pressure transmitter	0–1 bar	Pressure drop, ΔP	$U_{\Delta P}=1\;\times \;\frac{0.4\%}{10\;K}$ × (90−20) ≅ ±0.028 bar	12.727%	-
4	Flowmeter	1–90 L/min	Volume flow rate, ∀˙	$U_{\forall }^{.}=\sqrt{{\left(0.1\right)}^{2}+{\left(0\right)}^{2}}$ ≅ ±0.1 L/min	0.04%	1.0%
5	Thermophysical properties	Thermal conductivity, *k* Dynamic viscosity, *µ* Density, ρ Specific heat, *C_p_*		$\frac{U_{k}}{k}=\sqrt{{\left(0.05\right)}^{2}+{\left(0.0354\right)}^{2}}=$ ±6.13% $\frac{U_{µ}}{µ}=\sqrt{{\left(0.05\right)}^{2}+{\left(0.0527\right)}^{2}}=$ ±7.26% $\frac{U_{\rho }}{\rho }=\sqrt{{\left(0\right)}^{2}+{\left(0.0019\right)}^{2}}=$ ±0.19% $\frac{U_{C_{p}}}{C_{p}}=\sqrt{{\left(0\right)}^{2}+{\left(0.025\right)}^{2}}=$ ±2.5%	0.19%	7.26%

**Table 7:** Uncertainty of results calculated.

No	Result	Maximum Uncertainty
1	Mass flow rate, $\dot{m}$ = $\rho \dot{\forall }$	$\frac{U_{\Delta T}}{\Delta T}$ = ${\left[{\left(\frac{\partial \dot{m}}{\partial \rho }.\frac{U_{\rho }}{m}\right)}^{2}+{\left(\frac{\partial \dot{m}}{\partial \dot{\forall }}.\frac{U_{\dot{\forall }}}{\dot{m}}\right)}^{2}\right]}^{0.5}=$ [(0.03%)^2^ +(1.0%)^2^]^0.5^ = 1.00%
2	Temperature difference of fluid from inlet to outlet, ΔT = T_out_ − T_in_	$\frac{U_{\Delta T}}{\Delta T}$ = ${\left[{\left(\frac{\partial \Delta T}{{\partial T}_{out}}.\frac{U_{T_{out}}}{\Delta T}\right)}^{2}+{\left(\frac{\partial \Delta T}{{\partial T}_{in}}.\frac{U_{T_{in}}}{\Delta T}\right)}^{2}\right]}^{0.5}$ = ${\left[{\left(\frac{1.02}{5.5}\right)}^{2}+{\left(\frac{1.02}{5.5}\right)}^{2}\right]}^{0.5}$= 26.23%
3	Heat transfer, $\dot{Q}$ = $\dot{m}$ *c_p_*Δ*T*	$\frac{U_{\dot{Q}}}{\dot{Q}}\;$= ${\left[{\left(\frac{\partial \dot{Q}}{\dot{\partial m}}.\frac{U_{\dot{m}}}{\dot{Q}}\right)}^{2}+{\left(\frac{\partial \dot{Q}}{{\partial C}_{p}}.\frac{U_{C_{p}}}{\dot{Q}}\right)}^{2}+{\left(\frac{\partial \dot{Q}}{\partial \Delta T}.\frac{U\Delta T}{\dot{Q}}\right)}^{2}\right]}^{0.5}$ = ${\left[{\left(1.0\right)}^{2}+{\left(0.1\right)}^{2}+{\left(26.23\right)}^{2}\right]}^{0.5}$= 26.25%

## Data Availability

Data are available on request from the authors.
